# Wessex Branch of the Association of Clinical Pathologists

**Published:** 1988-11

**Authors:** 


### Wessex Branch of the Association of Clinical Pathologists

### Meeting at the Postgraduate Centre, Frenchay Hospital on 12 November 1987

**Under the Presidency of Professor P P Anthony (Exeter), the following abstracts of papers were amongst those delivered in the Scientific Session of the Autumn Meeting. Dr Elizabeth Mackenzie (Southmead) has now succeeded Dr K A V Cartwright (Gloucester PHLS) as the Branch Secretary. Congratulations were extended to Dr R J C Hart (Exeter), one time Education Secretary, on his election as Vice-President of the Association.**

### THE ROLE OF T8 Fc R+ LYMPHOCYTES IN A CASE OF CHRONIC T-CELL LYMPHOCYTOSIS AND NEUTROPENIA

**J G Smith, M A Smith*, I James**, E Blundell and P J Maddison***
Departments of Haematology and Immunology**, Royal United Hospital, Bath, and Royal National Hospital for Rheumatic Diseases, Bath*

A 45 year old Caucasian female with seropositive rheumatoid arthritis was found coincidentally to have a peripheral lymphocytosis (6.4×109/1) with neutropenia (1.0×109/1). Spleen scan showed no evidence of splenomegaly and serum titres against EBV, CMV and toxoplasmosis were negative. No anti-neutrophil antibodies were found. Immunoglobulin levels were normal and chromosome analysis of the blood lymphocytes showed a normal female karyotype. Marrow aspiration demonstrated a lymphocytosis of 60% with reduced numbers of granulocyte precursors. Phenotypic analysis with monoclonal antibodies revealed that both blood and marrow lymphocytes were T8, HLA-DR and Fc R positive. Approximately half the lymphocytes were weakly Leu 11 positive (mature NK marker). Lymphocyte conditioned medium (LCM) generated from the patient's blood lymphocytes (without phytohaemagglutinin) was found to be inhibitory for allogeneic CFU-gm stem cells in culture compared with control LCM. This inhibitory activity was abrogated by the cytolytic removal of the T8 cells prior to LCM production. Culture of the patient's marrow at autostimulatory light density marrow cell concentrations (5×105 cells/ml), showed poor spontaneous CFU-GM colony formation until the marrow T8 lymphocytes were removed cytolytically. Prednisolone therapy resulted in the patient's neutrophil count rising from 0.03×109/l to 1.1×109/l and resolution of her clinical symptoms.

The work highlights the inhibitory role of T8 Fc R+ lymphocytes in chronic T-cell lymphocytosis, the mechanism of the neutropenia in this case, being the production of the humoral inhibitory substance. Studies are underway to characterise the nature of this inhibitor.

### MEDULLOMYOBLASTOMA WITH DIVERGENT GLIAL DIFFERENTIATION IN AN ADULT

**J A R Nicoll**
Frenchay Hospital, Bristol

A case is presented of a cerebellar tumour in a 63 year old man. On light microscopic examination large areas of the specimen were shown to consist of primitive undifferentiated tumour with indistinct solid Homer Wright rosettes. The appearances were those of a typical medulloblastoma. However, other areas of the tumour had different appearances. Some areas had the appearance of astrocytoma with microcysts and cells producing processes which stained with Phospho Tungstic Acid Haematoxylin and also using the immunoperoxidase technique with antibody to Glial Fibrillary Acid Protein. Other areas had an appearance suggestive of oligodendroglioma, the cells having uniform rounded nuclei and perinuclear haloes. Yet other areas had the appearance of ependymoma with typical perivascular pseudorosettes. This medulloblastoma was therefore exhibiting differentiation along three different glial lines.

In addition, scattered round cells were present which had abundant eosinophilic cytoplasm. These cells were identified as rhabdomyoblasts by staining using the immunoperoxidase technique with antibody to myoglobin, and on electron microscopy structures resembling sarcomeres composed of alternating thick and thin filaments were demonstrated in the cytoplasm.

This tumour is unusual, firstly in the age of the patient, secondly in the prominent divergent glial differentiation, and thirdly in the presence of rhabdomyoblasts. The question is raised whether primitive neuroectodermal cells might be capable of differentiating into rhabdomyoblasts or whether this tumour should be regarded as a teratoma.

### THE RESPONSE OF UROTHELIUM TO CATHETERISATION

**T J Clarke**
Department of Histopathology, Derriford Hospital, Plymouth

Indwelling catheters in the urinary bladder can produce a localised roughening of the urothelium which at cystoscopy can mimic papillary transitional cell carcinoma. Cystoscopic biopsies were taken from the site of maximal catheter reaction and from normal urothelium in thirty consecutive catheterised patients at the time of transurethral prostatectomy. The duration of catheterisation varied from two days to three years and a variety of catheters had been used.

The catheter reaction was characterised by an inflammatory response rich in eosinophil polymorphs in 26 patients (86%) with formation of oedematous polyps in 5 patients (17%). Intra-epithelial eosinophilic microabscesses were found in 9 patients (30%), each catheterised for at least one month. Urothelial hyperplasia was present in 11 patients (36%). Neither urothelial necrosis nor biopsy sites were present in 26 patients (86%). There was no correlation between the presence or absence of proven urinary tract infection and the degree of inflammatory response.

Moderately severe urothelial dysplasia, confined to the catheter reaction site, was present in two patients (6%), each catheterised for at least four months. This finding is relevant to the genesis of carcinoma in the catheterised urinary bladder of spinal injury patients (the incidence is 10% following catheterisation for at least 10 years).

### CAN CLINICIANS STAGE RECTAL TUMOUR INVASION PREOPERATIVELY

**H Rigby, J Beynon, J L Channer, N J Mc Mortensen, J W B Bradfield**
Bristol Royal Infirmary

The surgical management of primary rectal tumours, including local excision and sphincter saving procedures is facilitated by a knowledge of both local tumour spread and lymph node involvement. The present method of assessment of digital examination is highly subjective. Per rectum endoluminal sonography has proved successful in the pre-operative assessment of rectal tumour invasion.

In a pilot study the sonographic characteristics of normal bowel wall were determined using resected specimens. By the sequential stripping, and scanning between each procedure, it was possible to recognise and identify the sonographic features of five layers which corresponded histologically with the mucosal surface, mucosa and muscularis mucosa, submucosa, muscularis propria, and perirectal fat.

Ninety-three patients with primary rectal tumours, of which 86 subsquently underwent tumour resection, were examined using endoluminal sonography. The maximum depths of tumour invasion detected by ultrasound were compared with maximum depths of invasion on histological section. Local invasive depth was predicted with an accuracy of 92% and correlated closely with histological depth ($r=0.88$ $p<0.001$). Prediction of extension beyond the muscularis propria was achieved with a sensitivity of 97%, a specificity of 89%, a positive predictive value of 97% and a negative predictive value of 89%.

Lymph node involvement was assessed in 79 patients and was correctly predicted in 33 out of 39. The absence of lymph node metastases was correctly predicted in 29 out of 40 patients. Comparison of pre-operative nodal staging with postoperative histology gave an overall correlation of 0.73 ($p<0.001$). The accuracy of the technique in predicting nodal metastases was 78%, the sensitivity 85%, the specificity 73%, the positive predictive value 75%, and the negative predictive value 83%.

A detailed study of the false positive nodes in eight patients (a total of 27 nodes) failed to identify any particular histological feature responsible for the false positivity.

### AN OUTBREAK OF MENINGOCOCCAL MENINGITIS IN A ROYAL NAVY TRAINING ESTABLISHMENT

**P N Gaunt**
Derriford Hospital, Plymouth

In the spring of 1987 there was an outbreak of group C meningococcal meningitis in a Royal Navy shore-based establishment for training naval recruits. Three trainees developed meningococcal meningitis within a week in mid-February. Posterior pharyngeal swabs (PPS) taken from 55 dormitory contacts revealed pharyngeal carriage of meningococci in 34 (62%), of which 24/34 (71%) were indistinguishable from the outbreak strain.

### MANAGEMENT OF THE OUTBREAK

The main objectives of outbreak management were to prevent further cases of meningococcal disease in the base and to limit the spread of the outbreak strain into the local community and to other naval establishments. PPS were taken from all personnel on the base to establish the extent of the outbreak, menigococcal A+C polysaccharide vaccine was offered to all personnel, and rifampicin 48 hours after completion of the course, from trainees on entering in subsequent weeks and also during their penultimate week of training.

Rifampicin failed to eradicate meningococci from the pharynx of 26% of recipients, and 10% of those initially clear had acquired carriage in one week. The major factor in failure of rifampicin was poor compliance for a quarter of trainees questioned admitted to not taking all four doses. In those who had taken the full course of rifampicin efficacy was over 90%. It was therefore decided to attempt eradication of the outbreak strain from the base by giving all personnel a single oral dose of 500 mg ciprofloxacin.

At the end of March all personnel received ciprofloxacin after a PPS was taken to identify carriers. Therapy failed in 13/336 (3.9%) carriers followed-up four days later. No strains resistant to ciprofloxacin were isolated before or after its use and the prevalence of group C menigococci in trainees had fallen to less than 1% by the end of spring term when control measures were stopped.

### CONCLUSION

This outbreak highlights the unsuitability of rifampicin and the efficacy of ciprofloxacin in reduction of pharyngeal carriage in large populations. We would recommend that future outbreaks of this kind be managed with the early concurrent administration of polysaccharide vaccine and ciprofloxacin to the entire population at risk.

### LYMPHOMA AND THE KIDNEY

**Judith L Channer and A G MacIver***
Bristol Royal Infirmary, *Southmead Hospital, Bristol

Although direct infiltration of renal parenchyma by lymphoma is often seen at post mortem, it rarely produces impairment of renal function. Four cases of non-Hodgkin's lymphoma, all with significant renal involvement, are presented. Two of these patients presented with acute renal failure of unknown cause and diagnosis of non-Hodgkin's lymphoma was first made on renal biopsy. Three out of the four cases had renal failure, the fourth had unilateral renal enlargement. In the cases with renal impairment we must assume the disease is bilateral. No other cause for the renal impairment, such as light chain nephropathy or uric acid nephropathy, was detected. In addition, no glomerular abnormality was present in any of the cases. Immunoperoxidase studies using MT1 and MB2 revealed that all four tumours marked as T cell lymphomas.

In one case, non-Hodgkin's lymphoma was confined to the kidneys, with no evidence of disease elsewhere, representing an example of a primary renal lymphoma. The possibility of a normal population of T cells within the kidney, "kidney associated lymphoid tissue" from which such tumours may arise is discussed.

Further studies using a panel of monoclonal antibodies are in progress.

### THE USE OF DNA MOLECULAR PROBES IN THE DIAGNOSIS OF LYMPHORETICULAR DISEASE

**N Rooney, J Russell, N Maitland, J Goepel, J W B Bradfield**
Universities of Bristol and Sheffield

Fifty-five lymphoreticular biopsies were analysed for clonal rearrangement of the immunoglobulin (Ig) and T antigen receptor (TcR) by southern blotting. Digests were performed with Bam HI, Bgl II, Eco R1 and Hind III; probes to JH, Kappa, lambda and TB were supplied by T Rabbitts.

Clonal rearrangements of Ig were documented in 21/22 nodal and 4/5 extra-nodal B cell lymphomas. One clonal rearrangement of TB was found in seven T cell proliferations. No clonal rearrangements were found in 10 cases of Hodgkin's disease or 11 reactive tissues.

RNA expression correlated with the immunohistorchemistry results and indicated good nucleic acid recovery. The technique is expensive and occasional aberrant resultsremain so its practical use in the majority of laboratories is questionable.

